# Evaluation of the effects of implementing an electronic early warning score system: protocol for a stepped wedge study

**DOI:** 10.1186/s12911-016-0257-8

**Published:** 2016-02-09

**Authors:** Timothy Bonnici, Stephen Gerry, David Wong, Julia Knight, Peter Watkinson

**Affiliations:** 1 Kadoorie Centre for Critical Care Research and Education, Level 3, John Radcliffe Hospital, Headley Way, Oxford, OX3 9DU UK; 2 Centre for Statistics in Medicine, University of Oxford, Botnar Research Centre, Windmill Road, Oxford, OX3 7LD UK; 3 Institute of Biomedical Engineering, University of Oxford, Old Road Campus Research Building, Oxford, OX3 7DQ UK

**Keywords:** Vital Signs, Early Warning Score, Track and Trigger, Electronic Charting, Stepped-Wedge

## Abstract

**Background:**

An Early Warning Score is a clinical risk score based upon vital signs intended to aid recognition of patients in need of urgent medical attention. The use of an escalation of care policy based upon an Early Warning Score is mandated as the standard of practice in British hospitals. Electronic systems for recording vital sign observations and Early Warning Score calculation offer theoretical benefits over paper-based systems. However, the evidence for their clinical benefit is limited. Previous studies have shown inconsistent results. The majority have employed a “before and after” study design, which may be strongly confounded by simultaneously occurring events. This study aims to examine how the implementation of an electronic early warning score system, System for Notification and Documentation (SEND), affects the recognition of clinical deterioration occurring in hospitalised adult patients.

**Methods:**

This study is a non-randomised stepped wedge evaluation carried out across the four hospitals of the Oxford University Hospitals NHS Trust, comparing charting on paper and charting using SEND. We assume that more frequent monitoring of acutely ill patients is associated with better recognition of patient deterioration.

The primary outcome measure is the time between a patient’s first observations set with an Early Warning Score above the alerting threshold and their subsequent set of observations. Secondary outcome measures are in-hospital mortality, cardiac arrest and Intensive Care admission rates, hospital length of stay and system usability measured using the System Usability Scale. We will also measure Intensive Care length of stay, Intensive Care mortality, Acute Physiology and Chronic Health Evaluation (APACHE) II acute physiology score on admission, to examine whether the introduction of SEND has any effect on Intensive Care-related outcomes.

**Discussion:**

The development of this protocol has been informed by guidance from the Agency for Healthcare Research and Quality (AHRQ) Health Information Technology Evaluation Toolkit and Delone and McLeans’s Model of Information System Success. Our chosen trial design, a stepped wedge study, is well suited to the study of a phased roll out. The choice of primary endpoint is challenging. We have selected the time from the first triggering observation set to the subsequent observation set. This has the benefit of being easy to measure on both paper and electronic charting and having a straightforward interpretation. We have collected qualitative measures of system quality via a user questionnaire and organisational descriptors to help readers understand the context in which SEND has been implemented.

**Electronic supplementary material:**

The online version of this article (doi:10.1186/s12911-016-0257-8) contains supplementary material, which is available to authorized users.

## Background

Worsening physiological observations have been repeatedly shown to precede adverse outcomes in the hospital patient population [1–3]. However, these physiological alterations may go unrecognised, resulting in treatment delay [4]. Delays in appropriate treatment are known to worsen outcomes in acutely ill patients [2, 5, 6].

The concept of an Early Warning Score (EWS) was developed to aid recognition of deteriorating patients and has been adopted internationally [7–9]. An EWS is a weighted scoring system based upon vital sign observations – heart rate, blood pressure, temperature, respiratory rate, oxygen saturation and conscious level. The higher the EWS, the more abnormal the patient’s vital signs. NICE recommends that an EWS should be accompanied by an institution-specific protocol which stipulates changes in monitoring frequency and clinical management based upon the score [6].

Despite their theoretical benefits, EWS systems have not consistently been shown to affect hospital length of stay or mortality [10, 11]. Previous work has suggested that EWS systems may be less effective than anticipated because, scores are incorrectly assigned to individual vital signs, EWS values are incorrectly calculated or the care is not escalated appropriately [12–14].

Electronic EWS systems have the potential to mitigate some of these flaws. An electronic EWS system can be designed to automatically assign correct scores to vital signs, compute the EWS and prompt appropriate action. Where included, a central dashboard displaying recent observations and summary scores may increase the oversight of junior colleagues, facilitating improvements in the standards of practice.

Studies of electronic EWS systems have shown inconsistent results [15–17]. Many have used “before and after” design methodologies, comparing data from periods several years apart, with little attempt to correct for confounding factors [18]. This study design is strongly discouraged by the Cochrane Effective Practice and Organisation of Care (EPOC) Group [19]. At present there is no robust evidence to support or refute the case for the introduction of electronic EWS systems.

This study has been designed to investigate the clinical effectiveness of an electronic EWS system, System for Electronic Notification and Documentation (SEND). SEND will be rolled out sequentially according to a stepped wedge design across wards within each of four hospital. This will allow comparison of the electronic system with the paper system taking into account confounding factors and controlling for the effect of time without some of the limitations of previous studies.

### System description

A full description of SEND has been published previously [20]. In common with the other electronic charting systems [17, 21, 22] it provides automatic calculation of the EWS, displays relevant advice from local Trust protocols and provides an overview of the EWS values of all patients in a clinical area.

**Fig. 1:**
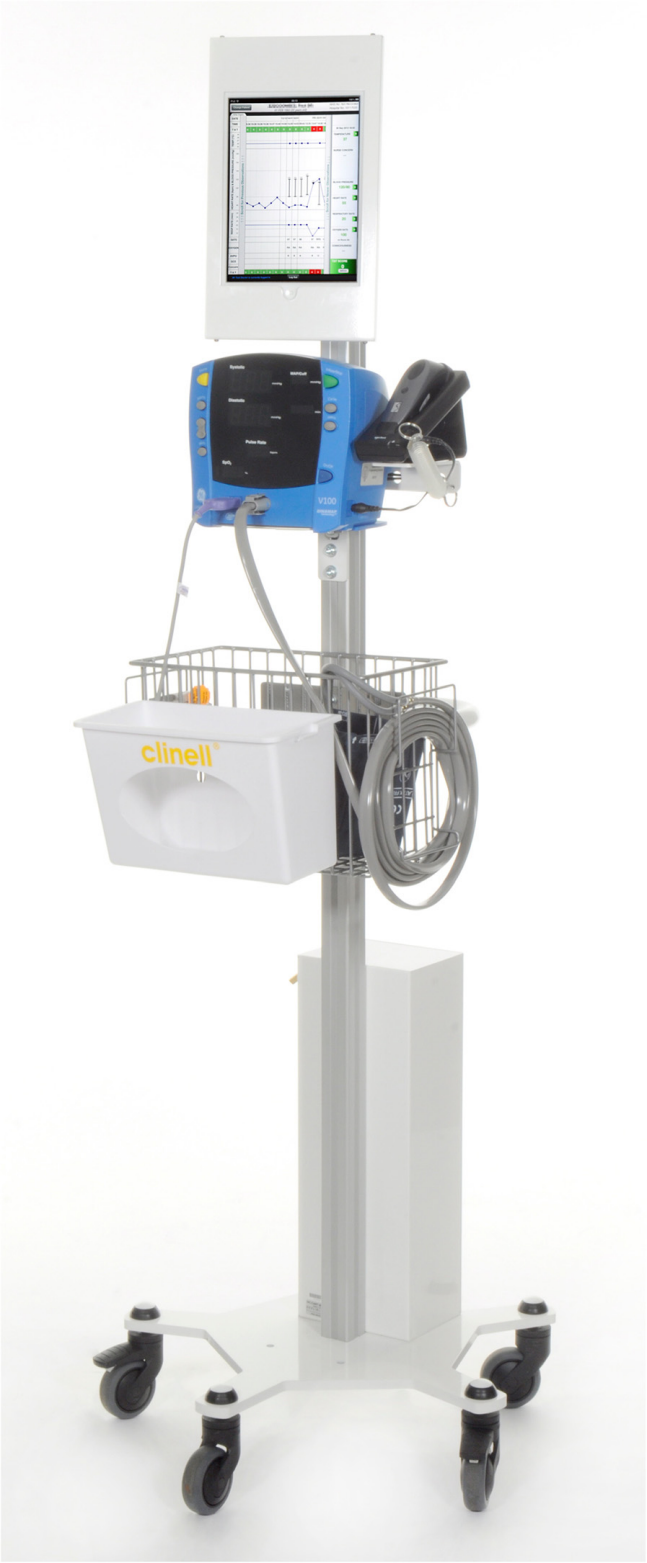
The SEND data entry hardware. Observation roll stand with equipment for taking observations, barcode scanner and tablet computer

**Fig. 2:**
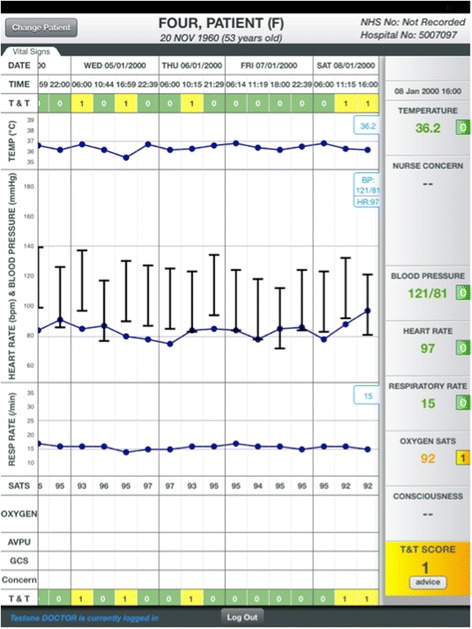
The chart viewing screen

Tablet computers used for data entry are mounted on the same stands as the physiological monitors used to measure patient vital signs (Fig. 1). This design is intended to minimise barriers to timely data entry and facilitate viewing of current and historical observations at the point of care using a graphical format that is familiar to clinical staff (Fig. 2). In order to minimise errors from misattribution of data, patients are identified by scanning their hospital wristband barcodes.

### SEND Implementation Plan

SEND will be used for the recording of routine observations on all adult wards and day units except for obstetrics wards and intensive care units (ICUs). A different EWS is used on obstetric wards and the ICUs already have an electronic charting system integrated into their patient record system.

**Fig. 3:**
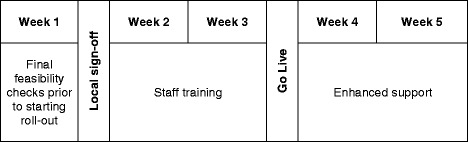
The implementation process for each ward

Deployment on each ward is a five week procedure, consisting of 3 weeks of preparation followed by two weeks of enhanced support following the date the system is activated on the ward. (Fig. 3) During the enhanced support weeks project staff are available on the ward to identify problems and offer additional training to users. In some cases it will be necessary to roll out groups of related wards simultaneously to ensure patient care is not compromised. Roll-out is planned to commence on a new ward (or group of wards) every fortnight. However, it is anticipated that practical difficulties may prevent this occurring in all cases. The sequence of ward roll-out is determined by pragmatic and patient safety considerations rather than being randomised.

As part of the implementation process ward managers are given access to a real-time audit report showing observation recording compliance at a ward level against Trust standards. This can be accessed at any time through the SEND application. A sample report is shown in the supplementary information. [Additional file 1].

## Methods

The study will use a non-randomised stepped wedge design carried out across the four hospitals of the Oxford University Hospitals (OUH) NHS Trust. A stepped wedge cluster design [23, 24] was chosen as it addresses the problem inherent in studying changes in process improvement [19].

A number of issues mitigate against the use of a randomised controlled trial. SEND must be simultaneously implemented for all patients within a ward. It is not removed once implemented in a study ward. Roll out must proceed sequentially across all wards. The order of ward roll out is dictated by operation factors and patient safety considerations therefore it is not be possible to randomise order in which the system is implemented in the study wards.

SEND will be implemented in all study wards in a stepwise fashion. Each time point at which a new study ward receives SEND marks the beginning of a new step. All study wards will have received SEND by the time point of the final step. The outcomes are measured in all study wards in each step. Observation recording practice will be assessed before and after the start of each step. The effect of time can be investigated and controlled for in the statistical analysis.

### Setting

The four hospitals include a district general hospital, Horton General Hospital, and three teaching hospitals (John Radcliffe Hospital, Churchill Hospital and Nuffield Orthopaedic Centre). The effects of implementation will only be studied on general wards (Level 0 and Level 1 wards as defined by the Intensive Care Society guidelines [25]). Day units, investigational units and higher dependency areas will not be studied. The Emergency Department and the Acute Medical Unit will also be excluded from study, as the manner in which these areas operate is substantially different from the remainder of hospital wards. The wards included in the study are hereafter referred to as ‘study wards’.

### Aim

The aim of this study is to determine whether introduction of SEND affects nursing recognition and response to signs of patient clinical deterioration.

### Hypothesis

The OUH NHS Trust has implemented the Centile-based Early Warning Score (CEWS) which defines at-risk patients as having a CEWS score of 3 or more [26]. Trust protocols mandate that a repeat observation of the vital signs should be performed within 1 h whenever a patient is at risk. Local audit suggests that these standards are not consistently met. Similar problems have been observed in other institutions [8, 22].

It is anticipated that SEND may affect the adherence to local policies on the frequency of observation recording for patients. As a result the observation frequency may change. A number of system features have the potential to contribute to this change. Integration of a tablet with vital signs monitors has previously been shown to reduce the time taken to record observations [27]. Preliminary data suggest that charting using SEND is faster than on paper. Lowering staff workload may improve observation frequency. SEND provides on-screen prompts and auditing of performance. Such measures have been shown to improve the frequency of observation recording [22]. However, staff are used to paper-based systems, which are readily accessible. Poor implementation of electronic vital signs systems has previously resulted in decreased charting rates [28].

Frequent patient observation is necessary but not sufficient to affect clinical outcome measures. The translation of improved management of deteriorating patients by nursing staff to better patient outcomes is dependent on the response of doctors as well as organisational facilities and processes – the “effector arm”. The implementation of SEND does not contain any measures designed to specifically improve the effector arm, therefore use of measures such as mortality or length of stay as the primary endpoint would not be suitable for assessing whether SEND improves nursing management. Nevertheless, these are clinically important measures and will be reported as secondary endpoints.

### Inclusion and exclusion criteria

The study period will commence two weeks prior to the roll out of SEND on the first ward in the initial hospital and finish three months after the roll out of SEND on the final ward in the final hospital. During this period all patients over the age of 16 years admitted to a study ward will be included, unless Trust protocol dictates that their observations should be recorded on a maternity observation chart. Admission episodes in which vital signs are charted on both paper and electronic systems during a single admission will also be excluded.

### Primary outcome measure

A patient is defined as being at risk of deterioration if they have a ‘triggering observation set’, one where the EWS ≥ 3. These patients should be observed at least hourly until the EWS falls below 3. The primary outcome measure is the time between a patient’s first triggering observations set and their subsequent set of observations. A shorter interval between observations is taken to represent improved response to potential deterioration.

To address potential confounding by patient length of ward stay, a triggering observation set will only be included in our analysis if it occurs within 48 h of admission to a study ward. If a patient has triggering observation sets on multiple study wards during the same admission episode only the first triggering set will be included in the analysis.

### Secondary endpoint measures

Secondary endpoints are: hospital mortality rate, ICU admission rate, cardiac arrest call rate, length of stay and usability as measured by the System Usability Scale (SUS) score. An increased ICU admission rate could be interpreted a marker of improved practice or worse practice. We hypothesise that earlier ICU admission will results in a shorter length of stay, lower ICU mortality and lower APACHE II acute physiology score on admission to ICU. These data will also be included in the secondary endpoints to aid interpretation of ICU admission rates.

### Data collection procedures

SEND will be rolled out in the four hospitals sequentially. Data collection will occur in a single hospital at any one time. At each site, collection of data from SEND will occur on all wards where SEND is being used for the duration of roll out. During a single admission episode patients will only contribute primary endpoint data from the first study ward to which they are admitted.

Collection of data from paper charts will occur on all wards where paper charts are being used. The time of first eligible triggering observations set and the time of the subsequent observations set will be collected by retrospective inspection of charts by a single assessor. Where no subsequent observations set exists the reason, where it can be inferred, will be noted (Table 1).

**Table 1:** Competing events which affect observation recording times

Competing Events
Cardiac arrest
Death
Discharge or transfer to another hospital
Medical emergency but not cardiac arrest
Palliation
Transfer to another ward
Transfer to ICU
Transfer to surgery

ICU-related data will be collected from the Intensive Care National Audit and Research Centre (ICNARC) database, length of stay and hospital mortality will be collected from the hospital Patient Administration System (PAS), cardiac arrest data will be collected from the Trust’s cardiac arrest audit database.

Time-varying covariates will be collected and included in the statistical model. These will include nurse staffing levels and the Standardised Hospital Mortality Ratio (SHMI) case-mix adjustment. In common with other software, the SEND application will be updated periodically to address issues raised by users and improve features. The release dates of new software versions will be recorded and may be included in the analysis. Other unanticipated events or changes in hospital process which are likely to impact on the performance of the SEND system will be documented if observed.

User feedback will be collected using a questionnaire consisting of the SUS questions supplemented by additional questions regarding the respondents’ demographics and experiences using the system [Additional file 2]. The SUS score is a validated measure of system usability [29].

Following completion of roll-out at a site all users at the site will be invited to complete the questionnaire. The questionnaire will be administered electronically. The questionnaire has been formatted following best practices [30]. Users will be given the opportunity to opt-out of receiving further emails. Except for those who have opted out, users who do not complete the questionnaires will be sent two reminders via email.

### Statistics

#### Analysis of outcome measures

A time to event analysis will be carried out to compare the primary outcome measure for paper-based charting with SEND-based charting. Competing events, such as transfer to theatre or cardiac arrest, may prevent a second set of observations being taken. Common electronically-recorded competing events are listed in Table 1. The time and type of any of these events will be recorded if they occur within 14 h of the triggering set of observations. If another set of observations or a competing event is not found within 14 h from the first triggering observation set it will be assumed that no further observations took place.

The time point of the step and ward will be included as covariates. The study design is multi-level, patients within ward, and a multi-level model will be used to take into account the correlation between patients within ward. Other covariates at the patient and ward level will be included in the models. A detailed statistical analysis plan will be written prior to analysis, however it is expected that the method for the primary outcome will be in the form of a semi-parametric random effects model, such as those described by Scheike et al. [31] and Zhou et al. [32].

Secondary endpoints will be reported for each period ward combination. A patient may be admitted to ICU more than once, but only the first ICU admission will be considered for the analysis. For each period-ward combination the probability of admission to ICU will be calculated from the number of patients admitted to ICU at least once and the total number of patients.

### Sample size

The number of patients included in the study will primarily be pragmatic. All eligible admissions will be included for the first hospitals. A subsequent interim analysis will determine what proportion of eligible admissions within a study ward in the paper charting cohort will be included for the final hospital, the largest of the four institutions. If the analysis determines that fewer than 100 % of admissions are required then admissions will be randomly selected. All eligible admissions where data is recorded using SEND will be included for all four hospitals. We anticipate that the total number of admissions included in the study will be around 60,000.

Power calculation for the first hospital is based upon analysis of (currently unpublished) data from a previous trial conducted locally. We assume that the proportion of patients who have a further observation within three hours of a recording an EWS ≥3 will be 0.5 in the paper arm (as seen in a previous study) and 0.6 in the electronic arm, that there will be an average of 11 patients with an initial EWS ≥3 per cell, and conservatively that the intra-cluster correlation (ICC) will be 0.15, the power is estimated to be 79.3 % for a 5 % alpha level.

This calculation depends on statistics estimated from the one small study. However it does indicate that when including all four hospitals the study will be sufficiently powerful to detect a difference of 10 % in the primary outcome between groups.

### Compensation for potential biases

#### Lack of randomisation

In a conventional stepped wedge randomised control trial design the order in which the clusters ‘switched’ treatment would be randomly chosen. Randomisation is not feasible here as the order and timing of roll out is dictated by operational factors and clinical risk. We do not consider this to be a significant limitation as it is unclear how a lack of randomisation in this design could manipulate results to favour either of the treatments.

#### Delayed Effect of intervention

It is possible that the effect of the intervention may increase as staff become more familiar with this system. Therefore the peak effect may be delayed for some weeks after roll out. This will reduce the power of the study. Additional follow-up time is a recognised method of compensating for this issue [33]. We will continue data collection for a further 3 months after completion of the last ward roll out at each site. Additional analyses will investigate whether the treatment effect changes with time from implementation of SEND.

#### Heterogeneity of patients between wards

Patients with similar characteristics are likely to be clustered within wards. Similarly wards of a similar type are clustered within the hospital sites. There is a risk that variation in outcomes between clusters may be large, limiting the inferences that can be made. The stepped-wedge design addresses this issue as it enables both between-cluster comparisons and within-cluster comparisons to be made. Further compensation will be provided by using ward level and patient level characteristics as covariates in the analysis.

#### External factors and time trends

External factors such as seasonal changes in workload may confound the outcomes. These are mitigated to some degree by the study design and data analysis which will include a time period covariate. The temporal variation of individual ward and patient level covariates can also be modelled.

#### Recurrent patient admissions

Patients may have more than one admission episode. For the purpose of the primary outcome the unit of analysis will be patient episodes rather than patients and we will treat multiple episodes within the same patient as independent. Timely recording of vital signs seems unlikely to be correlated with patient characteristics.

## Discussion

Measuring the effect of implementing a novel clinical information system is challenging. The practicalities of rolling out a system safely and effectively rarely permit a randomised study. The before-and-after design, which has been used frequently in the past, is subject to bias from confounding variables. We hope this study will provide a model for others to follow in the future.

The stepped-wedge design is well suited to the study of a phased roll-out. If properly analysed, adjusting for time and other confounding variables, the quality of evidence from a stepped wedge trial approaches that of a randomised controlled trial [34]. The best methods of modelling the data remain a subject for debate [35]. We will explore different approaches as part of our analysis.

The best metric to use for the primary endpoint is also challenging. Previous studies have used mortality and length of stay. We find it implausible that the introduction of a new charting system could significantly affect these endpoints alone. Length of stay, in particular, is affected by many external factors. Therefore we report these as secondary endpoints.

The incidence of admission to ICU is a measure related to patient deterioration but its interpretation is unclear. A rise in referrals may either indicate an increased awareness of patient deterioration or it may indicate an increased incidence of deterioration. It is also confounded by the fact that behavioural and systemic factors may inhibit admission to ICU, even if deterioration is recognised.

Our chosen metric, the time from the first triggering observation set to the subsequent observation set, has the benefit of being easy to measure on both paper and electronic charting systems. In contrast to ICU referral, the factors influencing the timing of the second set of observations are largely within the control of the attending nurse.

We have not chosen to measure the response to every triggering observation set as we anticipate that nursing behaviour will change to repeated alerts from an individual patient. Repeated alerting may indicate failure to adequately to treat deterioration but it may also reflect that some treatments take many hours to have an effect. In the latter situation gradually decreasing the frequency of observations may be appropriate.

We have designed our study based on the advice provided by the Agency for Healthcare Research and Quality (AHRQ) Health Information Technology Evaluation Toolkit [36]. This emphasises: (i) the benefits of mixed methods studies and (ii) the need to constrain the range of evaluation metrics to those deemed to be the most important.

We have chosen clinical effectiveness as the main focus of this study. However, it is not the only relevant outcome. Delone and McLeans’s Model of Information System Success [37] lists four determinants of success: system quality, information quality, service quality and user satisfaction. The user questionnaire will allow investigation of each of these aspects. In parallel to this trial we are conducting an investigation of the effects of SEND on nursing workload and a qualitative study of how SEND affects nurses’ ability to deliver care. These will be published separately.

Greenhalgh et al. [38] have highlighted that the effectiveness of a system cannot be divorced from the organisational and cultural setting in which it is implemented. For a description of the organisational culture within our hospitals we refer the reader to the work by Ovseiko et al. [39]. We will also provide descriptors of the hospitals and their case mix to allow readers to compare with their own institutions. By triangulating these sources of data we envisage that readers will be able to draw useful conclusions about how SEND might function in other environments.

## Ethics, consent and permissions

The study protocol was reviewed by the Trust’s Research and Development department and, based upon Healthcare Quality Improvement Partnership guidelines was deemed to be a service evaluation, not requiring review by the National Research Ethics Service. As patient data will be collected without their consent, permissions were obtained from Trust’s Caldicott Guardian and Medical Director in accordance with the Health Research Authority Confidentiality Advisory Group guidelines.

## Additional files

Additional file 1: **Sample audit report.** (PNG 781 kb)

Additional file 2: **The iSEND Study Questionnaire.** (PDF 51 kb)

## Abbreviations

APACHE II: acute physiology and chronic health evaluation ii
CEWS: centile-based early warning score
EWS: early warning score
ICNARC: intensive care national audit and research centre
ICU: intensive care unit
OUH: Oxford University Hospitals
PAS: patient administration system
SEND: system for electronic notification and documentation
SUS: system usability scale
