# Supplementary figures and images for: Evaluation of the effects of implementing an electronic early warning score system: protocol for a stepped wedge study
Source: BMC Med Inform Decis Mak. 2016 Feb 9;16:19. doi: 10.1186/s12911-016-0257-8 (PMC4748571; doi:10.1186/s12911-016-0257-8)

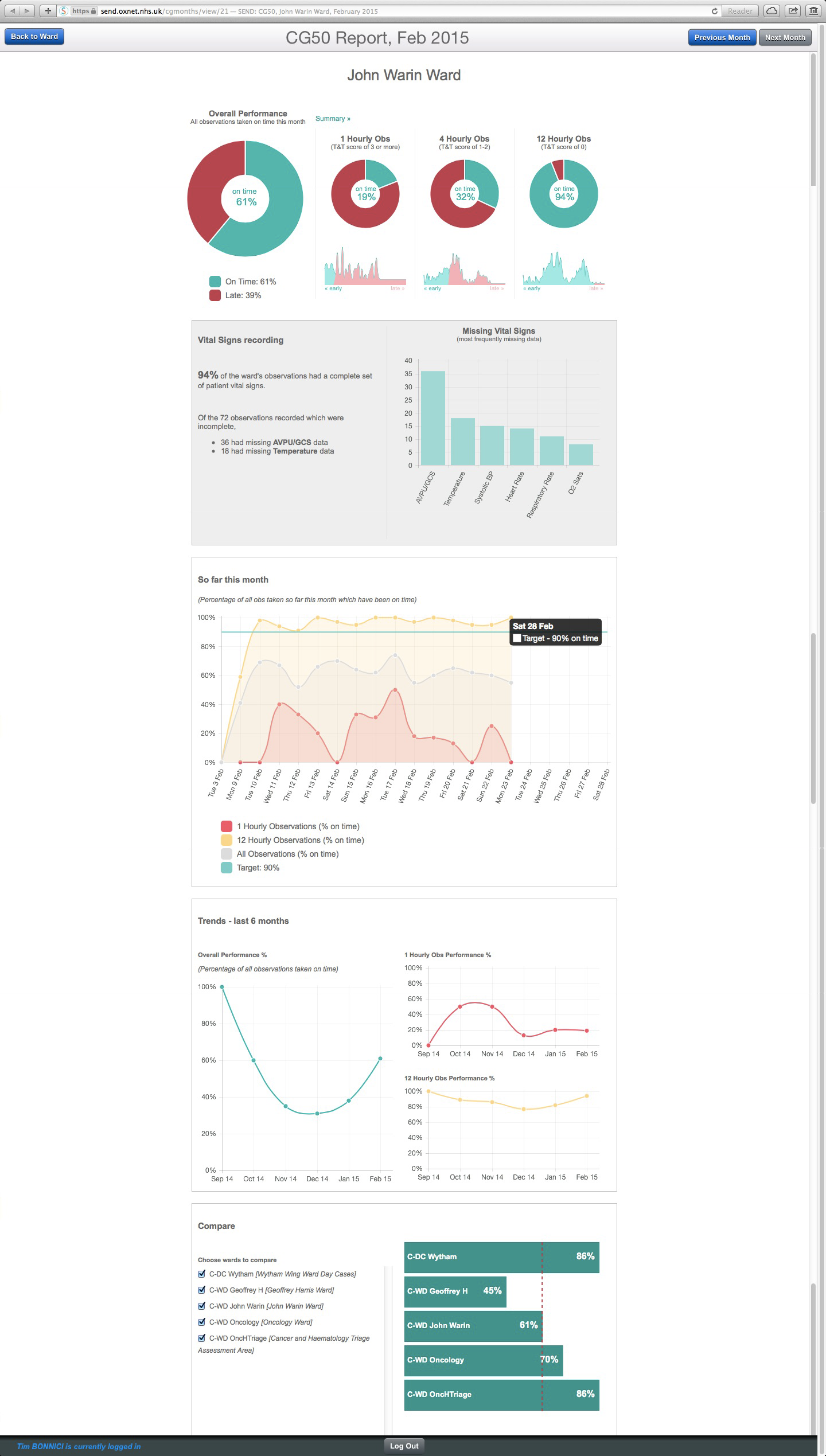

Supplement: Supplementary file 1 — Sample audit report. (PNG 781 kb) [file 12911_2016_257_MOESM1_ESM.png]
