# Supplementary material for: Evaluation of the effects of implementing an electronic early warning score system: protocol for a stepped wedge study
Source: BMC Med Inform Decis Mak. 2016 Feb 9;16:19. doi: 10.1186/s12911-016-0257-8 (PMC4748571; doi:10.1186/s12911-016-0257-8)
Supplement: Supplementary file 2 — The iSEND Study Questionnaire. (PDF 51 kb) [file 12911_2016_257_MOESM2_ESM.pdf]

iSEND Questionnaire

User Demographics

1. Please choose the job title which best describes your role.
   {Nurse/HCA, Doctor, Allied Health Professional, Non-clinical}
2. Please select the ward where you predominantly use the SEND application. If you work on multiple wards equally please choose the ward on which you first remember using SEND.
   {List of wards}
3. How many years have you worked within the NHS or equivalent healthcare system?
4. Do you feel that you have received adequate training in how to use SEND?

SUS Questions

Answered on a 5-point Likert scale ranging from Strongly Disagree to Strongly Agree

5. I think that I would like to use this website frequently.
6. I found this website unnecessarily complex.
7. I thought this website was easy to use.
8. I think that I would need assistance to be able to use this website.
9. I found the various functions in this website were well integrated.
10. I thought there was too much inconsistency in this website.
11. I would imagine that most people would learn to use this website very quickly.
12. I found this website very cumbersome/awkward to use.
13. I felt very confident using this website.
14. I needed to learn a lot of things before I could get going with this website.

Additional questions

15. Please describe how having SEND in your clinical area has affected the care you are able to deliver to patients.
16. Please describe any aspect of SEND training, installation or maintenance that has affected your ability to care for patients
17. Any other comments or suggestions?
18. If you would like us to respond to your comments directly please enter your email address. We will not use your email address for any other purpose.
